# Dibenzyl­aza­nium chloride

**DOI:** 10.1107/S1600536812003777

**Published:** 2012-02-04

**Authors:** N. Selvakumaran, R. Karvembu, Seik Weng Ng, Edward R. T. Tiekink

**Affiliations:** a Department of Chemistry, National Institute of Technology, Tiruchirappalli 620 015, India; b Department of Chemistry, University of Malaya, 50603 Kuala Lumpur, Malaysia; c Chemistry Department, Faculty of Science, King Abdulaziz University, PO Box 80203 Jeddah, Saudi Arabia

## Abstract

In the title salt, C_14_H_16_N^+^·Cl^−^, the complete cation and complete anion are generated by the application of mirror symmetry. The mol­ecule is nonplanar, as seen in the dihedral angle between the terminal phenyl rings [70.92 (5)°]. In the crystal, N—H⋯Cl hydrogen bonds involving both aza­nium H atoms link the ions into a zigzag supra­molecular chain along [100].

## Related literature

For the crystal structure of the isostructural bromide salt, see: Polamo *et al.* (1997 ▶).
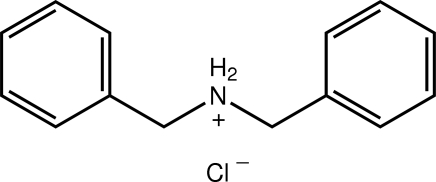


## Experimental

### 

#### Crystal data

C_14_H_16_N^+^·Cl^−^
*M* *_r_* = 233.73
Orthorhombic, 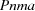

*a* = 10.1524 (9) Å
*b* = 23.8858 (17) Å
*c* = 5.0922 (4) Å
*V* = 1234.85 (17) Å^3^
*Z* = 4
Mo *K*α radiation
μ = 0.28 mm^−1^
*T* = 100 K
0.25 × 0.25 × 0.15 mm

#### Data collection

Agilent SuperNova Dual diffractometer with an Atlas detector
Absorption correction: multi-scan (*CrysAlis PRO*; Agilent, 2010 ▶) *T*_min_ = 0.933, *T*_max_ = 0.959
3840 measured reflections
1449 independent reflections
1092 reflections with *I* > 2σ(*I*)
*R*_int_ = 0.046

#### Refinement

*R*[*F* ^2^ > 2σ(*F* ^2^)] = 0.049
*wR*(*F* ^2^) = 0.125
*S* = 1.05
1449 reflections
82 parameters
H atoms treated by a mixture of independent and constrained refinement
Δρ_max_ = 0.37 e Å^−3^
Δρ_min_ = −0.23 e Å^−3^

### 

Data collection: *CrysAlis PRO* (Agilent, 2010 ▶); cell refinement: *CrysAlis PRO*; data reduction: *CrysAlis PRO*; program(s) used to solve structure: *SHELXS97* (Sheldrick, 2008 ▶); program(s) used to refine structure: *SHELXL97* (Sheldrick, 2008 ▶); molecular graphics: *ORTEP-3* (Farrugia, 1997 ▶) and *DIAMOND* (Brandenburg, 2006 ▶); software used to prepare material for publication: *publCIF* (Westrip, 2010 ▶).

## Supplementary Material

Crystal structure: contains datablock(s) global, I. DOI: 10.1107/S1600536812003777/hg5170sup1.cif

Structure factors: contains datablock(s) I. DOI: 10.1107/S1600536812003777/hg5170Isup2.hkl

Supplementary material file. DOI: 10.1107/S1600536812003777/hg5170Isup3.cml

Additional supplementary materials:  crystallographic information; 3D view; checkCIF report

## Figures and Tables

**Table 1:** Hydrogen-bond geometry (Å, °)

*D*—H⋯*A*	*D*—H	H⋯*A*	*D*⋯*A*	*D*—H⋯*A*
N1—H1n⋯Cl1	1.00 (4)	2.19 (4)	3.173 (2)	167 (3)
N1—H2n⋯Cl1^i^	0.99 (4)	2.16 (4)	3.104 (2)	160 (3)

Symmetry code: (i) 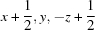.

**Fig. 1.**
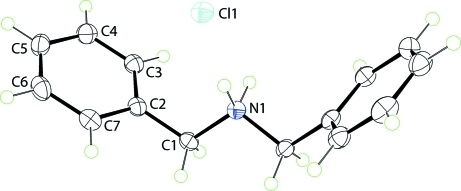
The molecular structures of the ions comprising (I) showing the atom-labelling scheme and displacement ellipsoids at the 50% probability level. The ions lie on a mirror plane and unlabeled atoms are related by x, 1/2 - y, z.

**Fig. 2.**
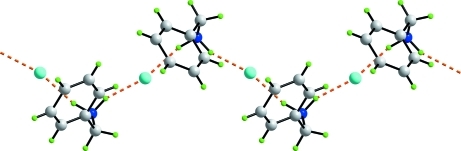
A supramolecular chain along [100] in (I) mediated by N—H···Cl hydrogen bonding shown as orange dashed lines.

## supplementary crystallographic information

### Comment

The title compound, (I), was obtained as an unexpected product from a reaction mixture containing dibenzylamine, isophthaloyl dichloride and potassium thiocyanate in acetone under reflux conditions, a reaction designed to form a thiourea derivative. Crystals were grown from a solution of the compound in ethylacetate / petroleum ether (1:3) mixture.

The NH_2_ atoms of the cation and Cl anion in (I), Fig. 1, lie on a crystallographic mirror plane. The dihedral angle between the symmetry related phenyl rings is 70.92 (5)°. Both ammonium-H atoms form hydrogen bonds to the Cl anion resulting in a supramolecular zigzag chains along [100], Fig. 2 and Table 1. Chains assemble into layers in the *ac* plane which stack along the *b* axis with no specific intermolecular interactions being present.

The structure of (I) is isostructural with the bromide salt (Polamo *et al.*, 1997).

### Experimental

A solution of isophthaloyl dichloride in acetone was added drop wise to a suspension of potassium thiocyanate in anhydrous acetone. The reaction mixture was heated under reflux for 45 minutes and then cooled to room temperature. A solution of dibenzylamine in acetone was added and the resulting mixture was stirred for 2 h. Hydrochloric acid (0.1 N, 300 ml) was added and the resulting white solid was filtered, washed with water and dried *in vacuo*. Single crystals were grown at room temperature from ethylacetate / petroleum ether (1:3) mixture.

### Refinement

The H-atoms were placed in calculated positions (C—H 0.95 to 0.99 Å) and were included in the refinement in the riding model approximation, with *U*_iso_(H) set to 1.2*U*_equiv_(C). The ammonium-H atoms were refined without restraint.

### Crystal data

**Table:**

C_14_H_16_N^+^·Cl^−^	*F*(000) = 496
*M**_r_* = 233.73	*D*_x_ = 1.257 Mg m^−^^3^
Orthorhombic, *P**n**m**a*	Mo *K*α radiation, λ = 0.71073 Å
Hall symbol: -P 2ac 2n	Cell parameters from 977 reflections
*a* = 10.1524 (9) Å	θ = 2.6–27.5°
*b* = 23.8858 (17) Å	µ = 0.28 mm^−^^1^
*c* = 5.0922 (4) Å	*T* = 100 K
*V* = 1234.85 (17) Å^3^	Prism, colourless
*Z* = 4	0.25 × 0.25 × 0.15 mm

### Data collection

**Table:**

Agilent SuperNova Dual diffractometer with an Atlas detector	1449 independent reflections
Radiation source: SuperNova (Mo) X-ray Source	1092 reflections with *I* > 2σ(*I*)
Mirror monochromator	*R*_int_ = 0.046
Detector resolution: 10.4041 pixels mm^-1^	θ_max_ = 27.6°, θ_min_ = 4.1°
ω scan	*h* = −10→13
Absorption correction: multi-scan (*CrysAlis PRO*; Agilent, 2010)	*k* = −30→27
*T*_min_ = 0.933, *T*_max_ = 0.959	*l* = −6→4
3840 measured reflections	

### Refinement

**Table:**

Refinement on *F*^2^	Primary atom site location: structure-invariant direct methods
Least-squares matrix: full	Secondary atom site location: difference Fourier map
*R*[*F*^2^ > 2σ(*F*^2^)] = 0.049	Hydrogen site location: inferred from neighbouring sites
*wR*(*F*^2^) = 0.125	H atoms treated by a mixture of independent and constrained refinement
*S* = 1.05	*w* = 1/[σ^2^(*F*_o_^2^) + (0.0542*P*)^2^ + 0.3264*P*] where *P* = (*F*_o_^2^ + 2*F*_c_^2^)/3
1449 reflections	(Δ/σ)_max_ < 0.001
82 parameters	Δρ_max_ = 0.37 e Å^−^^3^
0 restraints	Δρ_min_ = −0.23 e Å^−^^3^

### Special details

**Table:**

Geometry. All e.s.d.'s (except the e.s.d. in the dihedral angle between two l.s. planes) are estimated using the full covariance matrix. The cell e.s.d.'s are taken into account individually in the estimation of e.s.d.'s in distances, angles and torsion angles; correlations between e.s.d.'s in cell parameters are only used when they are defined by crystal symmetry. An approximate (isotropic) treatment of cell e.s.d.'s is used for estimating e.s.d.'s involving l.s. planes.
Refinement. Refinement of *F*^2^ against ALL reflections. The weighted *R*-factor *wR* and goodness of fit *S* are based on *F*^2^, conventional *R*-factors *R* are based on *F*, with *F* set to zero for negative *F*^2^. The threshold expression of *F*^2^ > σ(*F*^2^) is used only for calculating *R*-factors(gt) *etc*. and is not relevant to the choice of reflections for refinement. *R*-factors based on *F*^2^ are statistically about twice as large as those based on *F*, and *R*- factors based on ALL data will be even larger.

### Fractional atomic coordinates and isotropic or equivalent isotropic displacement parameters (Å^2^)

**Table:**

	*x*	*y*	*z*	*U*_iso_*/*U*_eq_	
Cl1	0.57091 (7)	0.2500	0.22464 (13)	0.0232 (2)	
N1	0.8159 (2)	0.2500	0.6115 (5)	0.0190 (5)	
C1	0.8343 (2)	0.30217 (8)	0.7718 (4)	0.0209 (5)	
H1A	0.9290	0.3068	0.8128	0.025*	
H1B	0.7862	0.2982	0.9398	0.025*	
C2	0.7854 (2)	0.35355 (8)	0.6298 (4)	0.0203 (4)	
C3	0.8515 (2)	0.37583 (8)	0.4155 (4)	0.0232 (5)	
H3	0.9302	0.3587	0.3547	0.028*	
C4	0.8031 (2)	0.42314 (9)	0.2893 (4)	0.0267 (5)	
H4	0.8485	0.4382	0.1422	0.032*	
C5	0.6883 (2)	0.44844 (9)	0.3782 (4)	0.0296 (5)	
H5	0.6551	0.4807	0.2916	0.035*	
C6	0.6222 (2)	0.42665 (9)	0.5932 (4)	0.0297 (5)	
H6	0.5438	0.4440	0.6545	0.036*	
C7	0.6708 (2)	0.37950 (9)	0.7183 (4)	0.0245 (5)	
H7	0.6254	0.3647	0.8660	0.029*	
H1n	0.730 (4)	0.2500	0.515 (7)	0.043 (10)*	
H2n	0.882 (4)	0.2500	0.468 (6)	0.039 (9)*	

### Atomic displacement parameters (Å^2^)

**Table:**

	*U*^11^	*U*^22^	*U*^33^	*U*^12^	*U*^13^	*U*^23^
Cl1	0.0175 (4)	0.0287 (4)	0.0235 (4)	0.000	−0.0025 (3)	0.000
N1	0.0158 (12)	0.0218 (12)	0.0194 (12)	0.000	−0.0001 (10)	0.000
C1	0.0218 (11)	0.0208 (10)	0.0200 (10)	−0.0022 (8)	−0.0015 (8)	−0.0033 (8)
C2	0.0185 (10)	0.0206 (9)	0.0217 (10)	−0.0014 (8)	−0.0034 (8)	−0.0040 (8)
C3	0.0217 (11)	0.0243 (10)	0.0236 (10)	−0.0008 (8)	−0.0020 (8)	−0.0032 (8)
C4	0.0290 (12)	0.0247 (11)	0.0264 (11)	−0.0024 (9)	−0.0007 (9)	0.0004 (9)
C5	0.0342 (13)	0.0228 (10)	0.0317 (12)	0.0050 (9)	−0.0082 (10)	−0.0031 (9)
C6	0.0224 (11)	0.0318 (11)	0.0349 (12)	0.0058 (10)	0.0002 (10)	−0.0081 (10)
C7	0.0218 (11)	0.0271 (11)	0.0247 (10)	−0.0041 (8)	0.0021 (9)	−0.0042 (9)

### Geometric parameters (Å, º)

**Table:**

N1—C1	1.501 (2)	C3—C4	1.390 (3)
N1—C1^i^	1.501 (2)	C3—H3	0.9500
N1—H1n	1.00 (4)	C4—C5	1.389 (3)
N1—H2n	0.99 (4)	C4—H4	0.9500
C1—C2	1.508 (3)	C5—C6	1.386 (3)
C1—H1A	0.9900	C5—H5	0.9500
C1—H1B	0.9900	C6—C7	1.385 (3)
C2—C3	1.387 (3)	C6—H6	0.9500
C2—C7	1.394 (3)	C7—H7	0.9500
			
C1—N1—C1^i^	112.2 (2)	C2—C3—C4	120.3 (2)
C1—N1—H1n	112.1 (9)	C2—C3—H3	119.8
C1^i^—N1—H1n	112.1 (9)	C4—C3—H3	119.8
C1—N1—H2n	108.5 (10)	C5—C4—C3	120.0 (2)
C1^i^—N1—H2n	108.5 (10)	C5—C4—H4	120.0
H1n—N1—H2n	103 (3)	C3—C4—H4	120.0
N1—C1—C2	111.96 (17)	C6—C5—C4	120.0 (2)
N1—C1—H1A	109.2	C6—C5—H5	120.0
C2—C1—H1A	109.2	C4—C5—H5	120.0
N1—C1—H1B	109.2	C7—C6—C5	119.8 (2)
C2—C1—H1B	109.2	C7—C6—H6	120.1
H1A—C1—H1B	107.9	C5—C6—H6	120.1
C3—C2—C7	119.18 (19)	C6—C7—C2	120.7 (2)
C3—C2—C1	122.01 (19)	C6—C7—H7	119.7
C7—C2—C1	118.81 (18)	C2—C7—H7	119.7
			
C1^i^—N1—C1—C2	−166.69 (13)	C3—C4—C5—C6	0.2 (3)
N1—C1—C2—C3	−71.7 (2)	C4—C5—C6—C7	−0.2 (3)
N1—C1—C2—C7	108.7 (2)	C5—C6—C7—C2	−0.2 (3)
C7—C2—C3—C4	−0.6 (3)	C3—C2—C7—C6	0.6 (3)
C1—C2—C3—C4	179.77 (18)	C1—C2—C7—C6	−179.77 (18)
C2—C3—C4—C5	0.2 (3)		

Symmetry code: (i) *x*, −*y*+1/2, *z*.

### Hydrogen-bond geometry (Å, º)

**Table:**

*D*—H···*A*	*D*—H	H···*A*	*D*···*A*	*D*—H···*A*
N1—H1*n*···Cl1	1.00 (4)	2.19 (4)	3.173 (2)	167 (3)
N1—H2*n*···Cl1^ii^	0.99 (4)	2.16 (4)	3.104 (2)	160 (3)

Symmetry code: (ii) *x*+1/2, *y*, −*z*+1/2.
